# Non-Destructive Surface Characterization Using Microscopic Imaging and Data Modeling

**DOI:** 10.3390/ma18184376

**Published:** 2025-09-19

**Authors:** Mariusz Mączka, Maciej Kusy, Anna Szlachta, Ewa Korzeniewska

**Affiliations:** 1 Department of Electronics Fundamentals, Rzeszow University of Technology, 35-959 Rzeszow, Poland; mmaczka@prz.edu.pl (M.M.); mkusy@prz.edu.pl (M.K.); 2 Department of Metrology and Diagnostic Systems, Rzeszow University of Technology, 35-959 Rzeszow, Poland; annasz@prz.edu.pl; 3 Institute of Electrical Engineering Systems, Lodz University of Technology, Stefanowskiego 18, 92-537 Lodz, Poland

**Keywords:** 3D surface reconstruction, digital image processing, image-based modeling, surface profile prediction

## Abstract

This article presents a novel method for converting a digital image of a conductive surface into its three-dimensional spatial representation. The developed approach utilizes a mathematical transformation of pixel intensity to the height value of the represented point. The method includes interpolation, automatic image segmentation, and predictive reconstruction of surface profiles, which significantly improves the quality of material surface representation. The method was implemented in a 3D model of a conductive structure created in the physical vacuum deposition method, and its capabilities were demonstrated using examples of simulations of the electric field distribution within and on the surface of the tested sample.

## 1. Introduction

Material surface properties are important for a wide range of engineering applications, influencing the functional characteristics of products. In the case of textile and metallic materials, the micro- and macrostructure of the surface determines parameters such as the ability to reflect or absorb light [1], abrasion resistance [2] and adhesion of functional layers [3,4,5]. Classical approaches utilize statistical descriptions of topography based on roughness parameters described in the [6,7] standards. However, these approaches have a limited ability to represent irregular or anisotropic surface features. In engineering practice, a realistic digital representation of these surfaces is also becoming increasingly important, both for the simulation of technological processes and for functional and esthetic design.

Modeling the surfaces of fabrics and metals is a challenge due to their significantly different structural and mechanical properties. Fabrics are characterized by anisotropic fiber structures, which influence local changes in roughness and elasticity [8,9]. Metal surfaces, in turn, especially after machining, polishing, or sandblasting processes, exhibit specific topographic profiles and micron-scale deviations that must be modeled both geometrically and functionally [10,11]. A common challenge remains the precise representation of roughness parameters such as R_a_, R_z_, and R_t_, as well as their impact on the physico-mechanical and tribological properties of materials [12,13,14,15].

The literature on textiles includes geometric models based on yarn and weave analysis [16,17,18], which take into account both physical texture and microscopic structure [19,20,21]. Additionally, simulation tools are being developed to model surface deformation under the influence of mechanical stress [22,23,24,25,26].

For metallic surfaces, models that account for the effects of machining as milling [27], grinding [28], and anodizing [29] are particularly important. Advanced analyses often utilize 3D topography data obtained using laser scanning microscopy (LSM) [30], atomic force microscopy (AFM) [31,32], and white light interferometry [33,34]. These models are used, among others, in predicting tool life, testing coating adhesion, and assessing optical characteristics [35,36,37,38,39].

In recent years, machine learning methods have been increasingly used to predict roughness parameters based on microscopic images [40,41] or sensory data [42,43]. Bidirectional reflectance distribution function (BRDF) models are being developed in computer graphics to describe how light reflects from surfaces [44,45,46]. An integrated approach combining empirical data with predictive capabilities that can be applied either to fabrics or metallic surfaces is lacking. This work aims to fill this gap.

Modeling textronic structures (MSTs) are a crucial research element that drive advances in design and enhances our understanding of the phenomena occurring within these structures. Our models enable analysis of field distribution and current flow in two and three geometric dimensions [47,48]. One of the most challenging tasks in the MST creation process is describing and representing the conductive path surface, which can be complex due to various substrates and usage effects. While describing such surfaces using analytical functions leads to efficient computational algorithms, it does not always accurately reflect their nature. Therefore, we have proposed a new approach involving the analysis and conversion of digital images of conductive surfaces into three-dimensional spatial representations.

Digital image analysis is widely used in science and engineering, especially in surface reconstruction and topography analysis. There are many known methods for converting images into numerical representations of profiles. One of the simplest approaches is direct mapping of pixel intensity to height values [49,50,51].

In this paper, we present a method for converting images into numerical surface profiles by mathematically transforming pixel intensities into height values for the corresponding points on the surface. This process involves converting the image to a numerical matrix, normalizing the pixel values, transforming the matrix into a height map, and extracting the surface profiles. Artificial intelligence methods are employed to improve the mapping quality through interpolation, automatic image segmentation, and predictive profile reconstruction.

The surface characterization method presented in this article applies to textile electronic structures (TSs), which are textiles integrated with electronic or conductive components. In this case, the conductive layer was produced using physical vapor deposition (PVD), a process in which a solid material is vaporized and deposited on the textile surface in a vacuum, creating a thin metal layer. This layer is conductive, meaning it allows electric current to flow. For electric field calculations, the area surrounding the conductive structure is treated as a dielectric, meaning it influences the electric field distribution without conducting electricity. The digital image conversion process in the presented method involves converting compressed pixel values into a numerical matrix. This matrix forms the basis for the subsequent transformation of intensity into height values, creating a numerical model of the surface.

## 2. Materials and Methods

**Figure 1:**
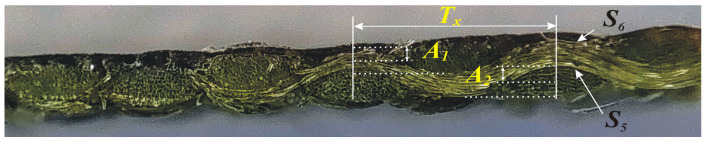
Illustrative microscopic cross-section of a TS sample (Cordura) made using the PVD method, with marked geometric parameters serving as the basis for the numerical model.

The structural modeling in this article is based on the example of a textile composite, Cordura. A microscopic cross-section of this product (see Figure 1) reveals a regular weave, in which the warp and weft yarns are arranged alternately, creating a characteristic weave with significant height differences between the yarn peaks and their intersections. The yarns have cross-sections that are close to circular or slightly flattened, tightly arranged, with minimal spaces between the elementary fibers. The outer surfaces of the yarns are visibly smooth. There are no protruding elementary fibers or gaps. Smooth material transitions are observed in the areas between the yarns, which may indicate partial filling of micro-gaps. The cross-section shows that the spaces between the yarns within the weave are not empty. The small defects, which are localized in the form of micropores or microcracks, occur particularly in areas of greater curvature, which may be the result of stresses generated during fabrication or sample preparation for observation.

The main peaks correspond to the cross-sections of the yarns, arranged in an alternating pattern. The surface curves over each yarn and then dips in the zones between them. The amplitude of this wave is clear and relatively large in relation to the wavelength.

At the yarn peaks, the surface contour is smooth, without sharp edges or significant bulges of the monofilaments, suggesting the presence of a thin, adherent layer of smoothing material.

Based on the above observations, parameters were defined that allowed for an approximate description of the geometry of the conductive surface produced on the material. These parameters include *A*_1_ and *A*_2_, which correspond to the amplitude of the upper (*S*_6_) and lower (*S*_5_) surfaces, respectively, as well as *T_x_*, which corresponds to the period of the warp thread that forms the fabric. The silver layer is the substrate on which the silver was sputtered.

### 2.1. Mapping Methodology

The Image conversion into numerical surface profiles is achieved by mathematically transforming pixel intensity to the height value of the point it represents. This approach allows for precise modeling of surface structures based on the information contained in digital images. The processing process begins with an image representation as a discrete function, where the input digital image is modeled as a function of two variables representing pixel intensity:

(1) $$I:\{(x,y)|\;x\in \{0,1,\ldots ,L_{x}-1\},y\in \{0,1,\ldots ,L_{y}-1\}\}\to \{0,1,\ldots ,255\}\;,$$

where *I* (*x*,*y*) is the pixel intensity value at position (*x*,*y*) and 0 ≤ *I* (*x*,*y*) ≤ 255 for grayscale images (or another range, depending on the image’s bit depth). The variables *L_x_* and *L_y_* determine the resolution of the loaded image in the *x* and *y* directions, respectively.

The next step in the process presented here is converting the image into a numerical matrix. The image read function reads the .png file, decodes the compressed pixel values, and maps them into a numerical matrix $A\in {ℜ}^{L_{x}\times L_{y}}$ as follows:

(2) $$A(y,x)=I(x,y)\;x=0,\ldots ,L_{x}-1,\;y=0,\ldots ,\;L_{y}-1,$$

Matrix **A** is a direct numerical representation of the image in memory, sorted row-wise, so **A**(*y*,*x*) stores *I*(*x*,*y*) indexed by the image height (*y*) and then by its width (*x*). The pixel values are then normalized. To obtain values in the range [0, 1], we apply normalization:

(3) $$A_{\mathrm{norm}}\;(y,x)=A(y,x)/255,$$

for *y* and *x* running along the height and width image indices, which ensures that all values are constrained according to the norm, i.e., **A**_norm_ (*y*,*x*) ∈ [0, 1]. In the next step, we calculate the height map. The surface height is mapped from the intensity values according to the following relationship:

(4) $$H(y,x)=A_{\mathrm{norm}}\;(y,x)=I(x,y)/255,$$

where *H* (*y*,*x*) ∈ [0, 1] denotes the given height of the surface, which assumes:

- if *I*(*x*,*y*) → 0 then *H*(*y*,*x*) → 0 what corresponds to darker pixels.
- if *I*(*x*,*y*) → 255 then *H*(*y*,*x*) → 1 what corresponds to brighter pixels.

In this way, the transformation of grayscale intensity to height $H\in {\left[0,1\right]}^{L_{x}\times L_{y}}$ is achieved by a simple linear mapping. The result is a height field, which can be interpreted as a surface profile derived from the original image. The final step is surface profile extraction, which extracts individual profiles along the *x*-axis (width) of the image.

For discrete pixel positions *x* = {0, 1, …, *L_x_* − 1}, each row y of the height map *H* (*y*,*x*) represents a cross-section of the surface at height *y*. These profiles are stored as follows:

(5) $$p_{y}=[H(y,0),H(y,1),\ldots ,H(y,L_{x}-1)],$$

For example, if the row number is 23 (i.e., y = 23), then 1250 pixels are selected along the width of the image at that height. This creates a surface profile:

(6) $$p_{23}=[H(23,\;0),H(23,1),\ldots ,H(23,1249)]\in {ℜ}^{1250},$$

where each value ***H*** (23,*x*) represents the height obtained from the grayscale intensity at a given *x* = 0,…,1249. This means that the entire image provides an image height of a number of profiles whose height depends on the image intensity, while the length of each profile is equal to the image width. As a result, we obtain a three-dimensional grid of points representing the tested surface. This grid can be used in a numerical model of the structure, which will be described later.

### 2.2. Sample Application

**Figure 2:**
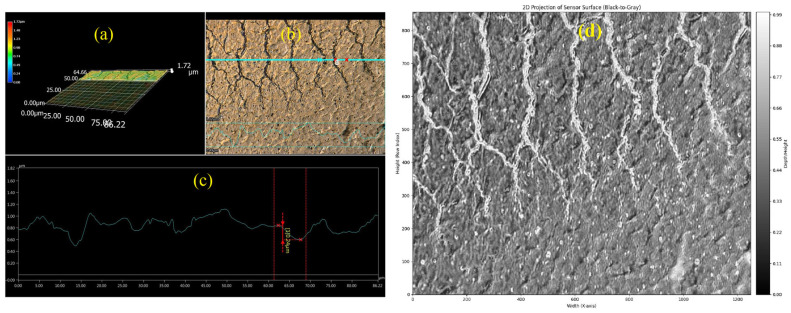
Microscopic images of the TS sample: (**a**) fragment of the surface map; (**b**) photo of the sample surface with the marked plane of the selected profilogram; (**c**) the profilogram along the marked cross-section of the sample; and (**d**) converted surface image to grayscale based on the amplitude of the profilogram visible in part (**c**).

The method described in the previous section was applied to surface images of a textronic structure taken with a Keyence VHX-7000 digital microscope (Keyence, Mechelen, Belgium), which provides high-resolution imaging and depth-composition capabilities. Figure 2 presents an example of such an image set. Part (a) shows the reconstructed three-dimensional topography of the scanned surface fragment, allowing the identification of micro-scale irregularities and height variations. Part (b) presents the top-down view of the same area with a marked cross-section line (in blue), along which the profilogram shown in part (c) was extracted. This profilogram clearly illustrates the amplitude and spatial frequency of the surface features in the selected direction, serving as a direct input for the grayscale conversion and height-mapping procedure described earlier. The accurate extraction and preprocessing of such profilograms are crucial, as any distortion or loss of detail at this stage would directly affect the fidelity of the numerical surface model and, consequently, the reliability of the subsequent electric field simulations.

The captured images not only document the physical surface condition but also serve as the primary dataset for subsequent numerical modeling, linking direct microscopic observation with computational field analysis.

The original microscope image, typically in RGB or color format (see Figure 2a,b), is converted to a single-channel grayscale image by computing a weighted sum of color components. Figure 2d illustrates the effect of this conversion, showing how the grayscale represents the depth of the surface.

**Figure 3:**
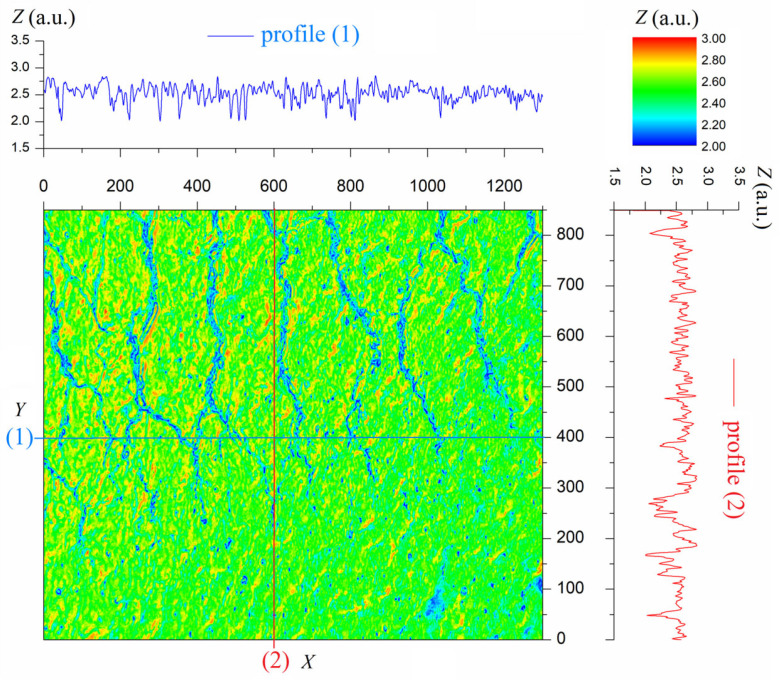
Surface topology of the tested TS sample in RGB color convention created by a point cloud obtained by analyzing a microscopic image, along with selected profiles in the x (blue graphs) and y (red graphs) directions.

Subsequent transformations described in the previous section result in a digital representation of the photo. From this representation, we can obtain a cloud of points that represent the nodes of the mesh that discretizes the examined surface of the structure, as shown in Figure 3.

**Figure 4:**
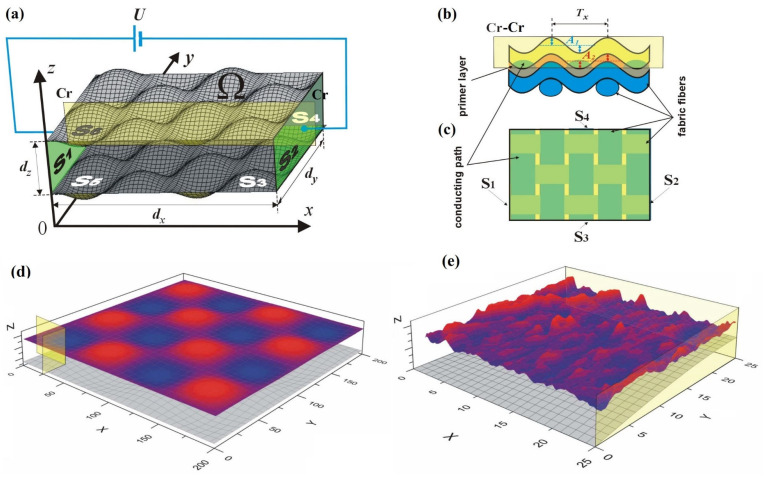
Three-dimensional geometric model of the textronic structure produced by PVD method on textile material boundary surfaces and voltage polarization system (**a**); view of the model in a cross-section with plane Cr (**b**) and top view of the model (**c**); illustration of the top surface generated using analytical function (**d**); and mapping method (**e**).

In this figure, a surface is plotted based on the *x*, *y*, and *z* coordinates of the points obtained from the image in Figure 2. Profiles 1 and 2 illustrate the surface roughness in the *x* and *y* directions, respectively. The obtained point cloud was incorporated into the previously developed 3D model of the textronic structure [23], which is illustrated in Figure 4a.

In this system, the points represent the top surface *S*_6_, generated using analytical formulas and shown in Figure 4b:

(7) $$f(\Omega ):\left\{\begin{array}{c} 0\leq x\leq d_{x}\\ 0\leq y\leq d_{y}\\ A_{2}(1+\sin k_{x}x\sin k_{y}y)\;\leq z\leq A_{1}(1+\sin k_{x}x\sin k_{y}y)+d_{z} \end{array}\right.,$$

This method provides an accurate representation of the actual topography of the top surface of the tested sample (see Figure 4c), but it requires additional procedures to match the geometric mesh of the newly derived top surface *S*_6_, to the geometric mesh of the other surfaces in the model. The shape of the top surface obtained using the new approach reveals sharp edges that could cause issues during numerical procedures for solving transport and field equations. Due to possible numerical errors when calculating the tangent and normal vectors and their derivatives, replacing surface *S*_6_ with a point cloud requires scaling and smoothing. The scaling parameters can be obtained directly from the microscopic images shown in Figure 1. The surface smoothing method (LSQ-Least Squares) is based on fitting a plane using the least squares method to local mesh fragments [52,53].

### 2.3. Method of Calculating Stationary Fields

The integration of the point cloud from the surface image analysis with the remaining boundary surfaces of the model allowed for the definition of a 3D region in which test simulations of stationary field distributions were performed. To accomplish this, we solved the Laplace equation in the following form:

(8) Δφ=0,

under mixed boundary conditions, constant potentials were assumed on surfaces *S*_1_ and *S*_2,_ and their difference was defined by the voltage *U*:

(9) $${\left.\varphi \right|}_{S_{1}}=\varphi (0,y,z)=U,$$

(10) $${\left.\varphi \right|}_{S_{2}}=\varphi (d_{x},y,z)=0,$$

Assuming that the environment of the analyzed area is an ideal dielectric and that electric current flows continuously, the normal component of the current density vector on surfaces *S*_3_–*S*_6_ must be zero. Neumann conditions on these surfaces:

(11) $${\left.\frac{\partial \varphi }{\partial n}\right|}_{S_{i}}=0,\;i=3,4,5,6,$$

To solve the above-formulated problem, the Iterative Fundamental Solution Method (IFSM) was used [54,55]. This method involves calculating successive approximations of the desired potential function according to the following formula:

(12) $${\tilde{\varphi }}_{k}(\vec{r})={\tilde{\varphi }}_{k-1}(\vec{r})+\sum_{n=1}^{L_{f}}q_{k,n}F_{k,n}(\vec{r}),$$

where

*k*—iteration step number.

$\vec{r}=\left(x,\;y,\;z\right)$—any point belonging to the region Ω.

*q_k_*_,*n*_—approximating sum coefficients calculated based on boundary conditions.

$F_{k,n}(\vec{r})=\frac{1}{\left|\vec{r}-{\vec{r}}_{k,n}\right|}$—fundamental solutions of the Laplace equation.

${\vec{r}}_{k,n}$—singular points of fundamental solutions established in each iteration step outside the region.

*L_f_*—number of fundamental solutions considered in a single iteration step.

For *k* = 0, the following (initial approximation) was assumed:

(13) $${\tilde{\varphi }}_{0}(\vec{r})=U\left(1-\frac{x}{d_{x}}\right),$$

This function satisfies Equation (5) and boundary conditions (6) through (8) on surfaces *S*_1_ through *S*_4_, thereby speeding up the convergence of calculations. It is also an exact solution for a flat layer (*A*_2_ = 0). Regardless of the *q_k_*_,*n*_ coefficients’ values, each function described by Formula (8) satisfies Equation (5) exactly. The choice of singular points of the fundamental solutions outside the region Ω guarantees boundedness in this region. The remaining details of solving the Laplace equation using the above method and the convergence conditions were identical to those in the approach described in [23].

Based on the calculated potential distribution, the electric field strength distribution was calculated according to the relationship:

(14) $$\vec{E}=-\vec{\mathrm{grad}}\varphi$$

The calculations were carried out under the assumption that the tested area is filled with a homogeneous, isotropic, and linear conductor (*γ* = const.), that there are no unbalanced electric charges (*ρ* = 0), that the surroundings are an ideal dielectric, and that there is a constant voltage *U* between surfaces *S*_1_ and *S*_2_.

## 3. Results

Based on the conversion methods described in the previous sections, a software module was developed and tested within the framework of the PVD textronic structure model. Sample calculation results were obtained using the following parameter values (in arbitrary units): *d_x_* = *d_y_* = 10, *A*_2_ = *A* = 0.5, *T_x_* = *T_y_* = 1, and *U* = 100 (see Figure 4). Due to the linear nature of the problem, the proportions of the geometric and physical quantities considered are important; therefore, the presented results refer to dimensionless relative values. In the case of geometric dimensions, all parameters refer to the thickness of the layer d, while the potential values are calculated in relation to the zero potential of surface S_2_.

**Figure 5:**
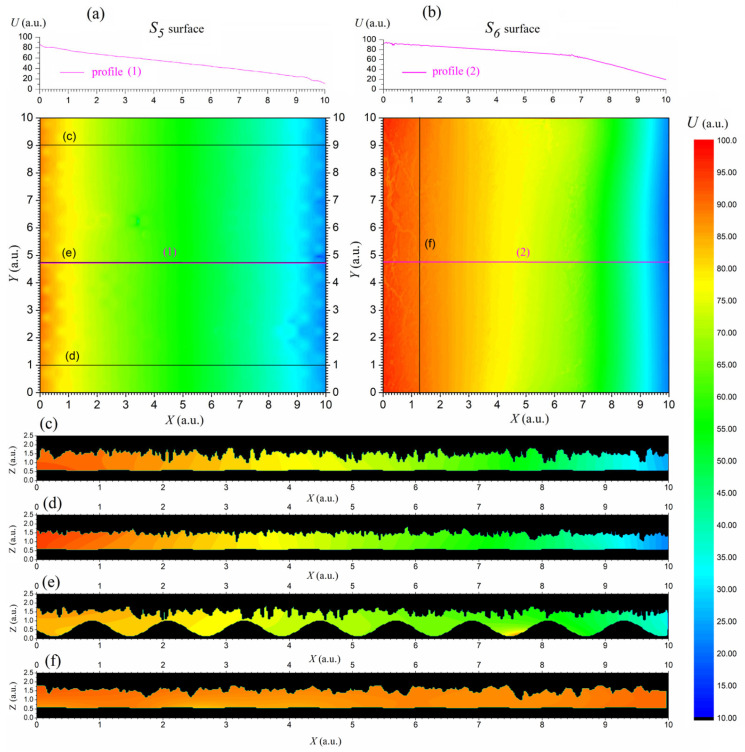
Results of solving the Laplace’s equation for the tested model of TS: potential on the XY surfaces of the lower S_5_ (**a**) and the upper S_6_ (**b**) surface with plotted profiles for locations (1) and (2), respectively, potential distribution maps in the XZ cross-sections (**c**–**f**), marked in figures (**a**) and (**b**) as (**c**), (**d**), and (**f**), respectively.

The integration of the reconstructed S_6_ surface with the sinusoidal S_5_ surface into the numerical model enabled the simulation of stationary electric field distributions by solving the Laplace equation under mixed boundary conditions. The left and right electrode surfaces (S_1_ and S_2_) were assigned constant but different potentials, while the side boundaries were treated as insulating. The calculation results for the test model defined in this way are presented in Figure 5.

Figure 5a,b illustrate the potential distribution on the lower (S_5_) and upper (S_6_) surfaces, respectively. The color maps show a clear potential gradient between the electrodes under voltage, which is consistent with the imposed voltage boundary conditions. Relationships can also be seen between the topography of the analytically described surface S_5_ and the pixel-height surface S_6_ and the associated potential distribution, which, respectively, exhibit periodic variations or reveal crack outlines. Profiles (1) and (2) highlight local fluctuations in the potential distribution resulting from surface roughness. These fluctuations are more visible on the lower surface described by the analytical formula (see profile (1) in Figure 5a) and slightly less pronounced on the upper surface, reconstructed based on microscopic imaging visible in Figure 2 (see profile (2) in Figure 5b). The potential field on this surface remains relatively homogeneous but shows visible deviations at the locations of material cracks.

Figure 5c–f show vertical cross-sections of the structure in the XZ plane, as indicated by the lines in Figure 5a,b. This visualization illustrates how the reconstructed geometry of the S6 surface affects the distribution of the internal potential. The waviness of the lower edge, combined with the irregular shape of the upper edge, leads to local field modulations visible as deviations from regular equipotential contours. The sharp edges of the upper surface can cause numerical problems or local intensification of the electric field. Therefore, the LSQ smoothing method was used, which eliminates computational problems while allowing the aforementioned field effects to be demonstrated.

**Figure 6:**
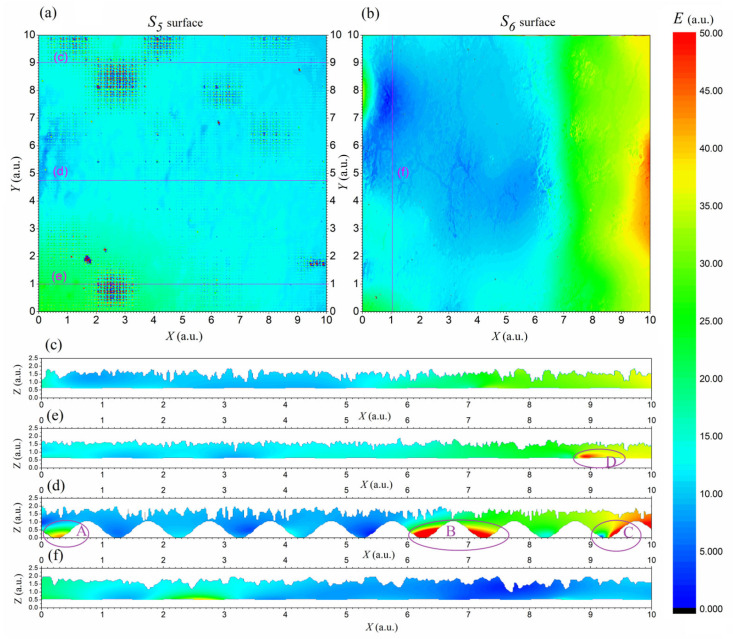
Simulation results of electric field intensity distributions on the XY surfaces of the lower S_5_ (**a**) and the upper S_6_ surface (**b**) and the field intensity distribution maps in the XZ cross-sections (**c**–**f**), marked in figures (**a**) and (**b**) as (**c**), (**d**), and (**f**), respectively.

Based on the calculated potential, a simulation of the electric field distribution was performed, and the results are illustrated in Figure 6. The top view (maps in Figure 6a,b) shows the field intensity on surfaces S_5_ and S_6_. As expected, the field is relatively uniform over large areas of the lower surface of S_5_, with local increases that are consistent with the nature of the analytical function that describes it. The upper surface also shows little change over a significant part of its area, although slight fluctuations can be observed near topographical irregularities. Areas of reduced intensity are clearly visible near depressions (cracks) and may correspond to locations where the potential gradient is smallest. This corresponds to the lower potential values visible in the same crack areas in Figure 5.

The cross-sectional graphs (Figure 6c–f) show the vertical distribution of the electric field intensity on selected XZ planes in the textronic structure. Both boundary surfaces, S_5_ (lower) and S_6_ (upper), have non-flat topography, which significantly affects the internal electric field. The lower surface (S_5_) is shaped by a periodic sinusoidal function in the X and Y directions, which causes regular, wave-like modulation of the lower edge in the X direction, particularly visible in cross-section (d). These undulations, combined with the irregular and spatially complex roughness of the upper surface S_6_, reconstructed from microscopic images in Figure 2, can introduce unpredictable changes in the local field strength due to large irregular changes in the thickness of the conductive path.

The undulation of the lower edge of the cross-section is not visible on the other cross-sections, because the course of lines (c), (e), and (f) falls within the areas between the vertices of the sine wave. This results in wider cross-sections of the conductive path, which can concentrate a higher electric field intensity in these cross-sections.

## 4. Discussion

As confirmed by the results presented in the previous section, the geometry of the interfaces significantly impacts the spatial distribution of the electric field in thin-film textronic structures. The two interfaces analyzed in the model, the lower S_5_ and the upper S_6_, both contribute to field nonuniformity, albeit in different ways.

The lower S_5_ interface is analytically defined by a two-dimensional sinusoidal function and introduces regular, predictable, periodic modulations of the electric field. These variations are visible in vertical sections as smooth, repetitive undulations of the field intensity. Although this function is mathematically idealized, it enables controlled testing of the model’s sensitivity to deterministic topography.

In contrast, the reconstructed top surface of S_6_, created from grayscale microscopic images, reveals highly irregular, non-periodic features that reflect the actual microstructure of the PVD-coated conductive layer. This surface produces sharp variations in local field intensity, especially near abrupt elevation changes. These effects are asymmetric and cannot be captured using standard analytical surface descriptions. This underscores the importance of incorporating empirical topographic data into simulation processes, particularly when evaluating phenomena like current densification, dielectric breakdown, and local heating in functional materials.

The use of a grayscale image-to-height mapping method proved effective in generating detailed topographical models from microscopy data. However, it also introduces numerical challenges: high local gradients can lead to poor mesh quality or unstable numerical derivatives, especially when computing normal vectors. To mitigate this, a surface smoothing procedure based on local least squares fitting (LSQ) was applied to the reconstructed point cloud. This step significantly improved the geometric compatibility of surface S6 with the remaining mesh and reduced numerical artifacts during simulation.

These findings support the broader conclusion that surface detail matters—even at the microscale—when modeling field-dependent phenomena in layered or printed electronic systems. The presented approach, combining empirical image-based modeling with numerical field simulation, creates new possibilities for analyzing realistic structures without relying solely on idealized assumptions.

An additional and practically significant observation can be made by analyzing the XZ cross-sections in Figure 6, particularly panels (d,e). In panel (d), numerous constrictions of the conductive gap are visible along the entire section; however, higher field intensities (warmer colors) occur only in selected locations, and predominantly closer to the lower surface S_5_. This indicates that the mere presence of a reduced gap is not sufficient to generate strong local fields. Instead, the observed distribution suggests that local field amplification depends on the combined effect of gap geometry, the spatial orientation of the surfaces relative to the applied potential direction, and the way the field lines are forced to converge or diverge by the topographic features. In panel (e), region D shows the opposite situation: a locally increased gap produces cooler colors (lower field) consistent with weaker potential gradients. The generally blue coloration of S_5_ in panel (a) further confirms that high field regions near the lower interface are localized and geometry-dependent rather than uniformly correlated with surface proximity.

It should also be noted that, given the numerical approach used—based on the method of fundamental equations with point sources placed outside the model boundaries—local field anomalies may partly reflect numerical artifacts. Factors such as the spatial distribution of the sources, the finite number of basic functions, and the reported RMS and maximum errors (10% and 30%, respectively) can introduce localized over- or underestimations of the field magnitude, particularly in areas with steep gradients or complex geometry. In the present study, the upper surface S_6_ mesh contained 1200 × 900 nodal points, directly corresponding to the pixel resolution of the analyzed microscope image. The lower surface S_5_ mesh was matched at 500 × 500 points, while the side surfaces (XZ and YZ) were discretized at 500 × 150 points. The number of point sources placed randomly at a distance of 0.7 au outside the model boundary in each iteration was set to be 10% greater than the number of nodal points along the corresponding model boundary. These parameters, while providing sufficient resolution for capturing the main geometric features, may still contribute to local inaccuracies in the most complex regions, underscoring the need for sensitivity analysis with respect to mesh density and source placement in future work.

Further simulations should be performed with systematically varying numerical parameters, including grid resolution, the number and spacing of point sources, and convergence tolerance, to confirm or rule out the numerical origin of the anomalies observed in regions A–D. Repeating the calculations with reduced RMS error targets (e.g., from 10% to less than 2%) and alternative source spacing strategies would help determine if the field peaks persist under more stringent numerical conditions. However, this is difficult, as achieving 10% convergence of the Laplace equation solution method took over 80 h of computation on an Intel Core i7+ machine using a Fortran programming language under Windows 11. Another approach is to perform a comparative analysis using an independent numerical technique, such as the finite element method (FEM). This could provide a benchmark for validating the fundamental equation approach in regions with complex geometry. However, this approach’s significant number of nodes can also cause numerical problems. In summary, only an efficient approach can make significant progress on this issue.

Another important limitation of the present study is its computational cost. In the current implementation, achieving 10% convergence required more than 80 h of calculations. While this is sufficient to demonstrate the method’s feasibility, such runtimes limit its broader applicability. Strategies for reducing computation time include adaptive mesh refinement, which focuses resolution on regions with high field gradients, parallelization of IFSM iterations, and GPU-based acceleration.

Additionally, the IFSM approach involves randomly placing field sources outside the modeled domain. Repeated runs of the algorithm with different random distributions may lead to variations in local field artifacts. This suggests that some anomalies could be reduced or relocated by optimizing or averaging multiple source configurations.

## 5. Conclusions

In this study, we presented a novel methodology for reconstructing the three-dimensional surface geometry of conductive materials using microscopic grayscale images. This approach allows for the non-destructive, image-based transformation of surface topography into a numerical model suitable for simulating electric field distributions.

Integrating an analytically defined lower surface (S5) and an empirically reconstructed upper surface (S6) demonstrated the combined influence of regular and irregular surface features on the spatial distribution of the electric field in a textronic structure. Simulations showed that even microscale variations in surface height can lead to localized field intensifications, particularly in regions where surface protrusions align or where geometric constraints focus the field lines. The analysis of XZ cross-sections (Figure 6) further revealed localized anomalies (regions A–D), which may originate from a combination of physical geometry effects and numerical artifacts, highlighting the importance of mesh design, source placement, and convergence control in such simulations.

In the present work, the approach was demonstrated on a Cordura-based textronic structure, as it was the only available sample at the time of the study. After successfully validating the numerical workflow with this case, the methodology can now be extended to other classes of materials. Future work will focus particularly on metallic surfaces with varying roughness levels and composite structures, where irregularities are expected to influence electrical performance significantly. The results of these studies will be presented in a separate publication.

Another limitation of the present study is the absence of a quantitative error analysis, such as an analysis of the root mean square (RMS) error distribution between the reconstructed and reference surfaces. Meaningful validation of this type would require comparison with precise profilometric measurements. To address this limitation, we have partnered with a research center that can provide high-resolution surface roughness data. This collaboration will enable us to more rigorously assess reconstruction accuracy in future work.

This methodology lays the groundwork for future research on textronic, printed, or flexible electronic devices, where the interaction between geometry and electrical behavior is crucial. Potential extensions include studies of transport parameters for different types of materials, optimization of surface mapping parameters, and cross-validation of results using alternative numerical techniques to confirm or exclude numerical contributions to localized field effects.

## Data Availability

The original contributions presented in this study are included in the article. Further inquiries can be directed to the corresponding author.

## Abbreviations

The following abbreviations are used in this manuscript:

BRDF	Bidirectional reflectance distribution function
LSM	Laser scanning microscopy
AFM	Atomic force microscopy
MSTs	Modeling textronic structures
